# Comprehensive Identification of Single Nucleotide Polymorphisms Associated with Beta-lactam Resistance within Pneumococcal Mosaic Genes

**DOI:** 10.1371/journal.pgen.1004547

**Published:** 2014-08-07

**Authors:** Claire Chewapreecha, Pekka Marttinen, Nicholas J. Croucher, Susannah J. Salter, Simon R. Harris, Alison E. Mather, William P. Hanage, David Goldblatt, Francois H. Nosten, Claudia Turner, Paul Turner, Stephen D. Bentley, Julian Parkhill

**Affiliations:** 1 The Wellcome Trust Sanger Institute, Wellcome Trust Genome Campus, Hinxton, Cambridge, United Kingdom; 2 Center for Communicable Disease Dynamics, Harvard School of Public Health, Boston, Massachusetts, United States of America; 3 Helsinki Institute for Information Technology HIIT, Department of Information and Computer Science, Aalto University, Espoo, Finland; 4 Department of Infectious Disease Epidemiology, Imperial College London, London, United Kingdom; 5 Immunobiology Unit, Institute of Child Health, University College London, London, United Kingdom; 6 Shoklo Malaria Research Unit, Mahidol-Oxford Tropical Medicine Research Unit, Faculty of Tropical Medicine, Mahidol University, Maesot, Thailand; 7 Centre for Tropical Medicine, Nuffield Department of Medicine, University of Oxford, Oxford, United Kingdom; 8 Cambodia-Oxford Medical Research Unit, Angkor Hospital for Children, Siem Reap, Cambodia; 9 Department of Medicine, University of Cambridge, Addenbrooke's Hospital, Cambridge, United Kingdom; University of Toronto, Canada

## Abstract

Traditional genetic association studies are very difficult in bacteria, as the generally limited recombination leads to large linked haplotype blocks, confounding the identification of causative variants. Beta-lactam antibiotic resistance in *Streptococcus pneumoniae* arises readily as the bacteria can quickly incorporate DNA fragments encompassing variants that make the transformed strains resistant. However, the causative mutations themselves are embedded within larger recombined blocks, and previous studies have only analysed a limited number of isolates, leading to the description of “mosaic genes” as being responsible for resistance. By comparing a large number of genomes of beta-lactam susceptible and non-susceptible strains, the high frequency of recombination should break up these haplotype blocks and allow the use of genetic association approaches to identify individual causative variants. Here, we performed a genome-wide association study to identify single nucleotide polymorphisms (SNPs) and indels that could confer beta-lactam non-susceptibility using 3,085 Thai and 616 USA pneumococcal isolates as independent datasets for the variant discovery. The large sample sizes allowed us to narrow the source of beta-lactam non-susceptibility from long recombinant fragments down to much smaller loci comprised of discrete or linked SNPs. While some loci appear to be universal resistance determinants, contributing equally to non-susceptibility for at least two classes of beta-lactam antibiotics, some play a larger role in resistance to particular antibiotics. All of the identified loci have a highly non-uniform distribution in the populations. They are enriched not only in vaccine-targeted, but also non-vaccine-targeted lineages, which may raise clinical concerns. Identification of single nucleotide polymorphisms underlying resistance will be essential for future use of genome sequencing to predict antibiotic sensitivity in clinical microbiology.

## Author Summary

*Streptococcus pneumoniae* is carried asymptomatically in the nasopharyngeal tract. However, it is capable of causing multiple diseases, including pneumonia, bacteraemia and meningitis, which are common causes of morbidity and mortality in young children. Antibiotic treatment has become more difficult, especially that involving the group of beta-lactam antibiotics where resistance has developed rapidly. The organism is known to be highly recombinogenic, and this allows variants conferring beta-lactam resistance to be readily introduced into the genome. Identification of the specific genetic determinants of beta-lactam resistance is essential to understand both the mechanism of resistance and the spread of resistant variants in the pneumococcal population. Here, we performed a genome-wide association study on 3,701 isolates collected from two different locations and identified candidate variants that may explain beta-lactam resistance as well as discriminating potential genetic hitchhiking variants from potential causative variants. We report 51 loci, containing 301 SNPs, that are associated with beta-lactam non-susceptibility. 71 out of 301 polymorphic changes result in amino acid alterations, 28 of which have been reported previously. Understanding the determinants of resistance at the single nucleotide level will be important for the future use of sequence data to predict resistance in the clinical setting.

## Introduction

Recent research aimed at finding the genetic causes of beta-lactam resistance in *Streptococcus pneumoniae* has been focused on laboratory mutagenesis [1]–[4], sequence comparison [1], [5], [6], and identification of interspecies sequence transfer that promotes penicillin non-susceptibility [7]–[10]. Though these studies have increased our understanding, their resolution is limited, and a whole-genome systematic search in real population settings is still lacking. Indicative of this limited resolution is the frequent use of the term “mosaic genes” to describe pneumococcal resistance alleles [7]. Although recombinational mosaics are clearly identifiable as regions of several hundred nucleotides in resistance genes, it is likely that only a subset of the observed alterations are important in causing resistance. Genome wide association studies (GWAS) have been used to identify genetic loci associated with complex diseases ranging from cancer to mental illness in human populations [11]–[13]. While the method should, in theory, be applicable to bacterial populations, its use has been inhibited by significant difficulties. These are primarily due to the clonal population structure, and generally limited recombination within bacteria, which make the causal SNPs indistinguishable from other linked SNPs, effectively creating very large haplotype blocks [14], [15]. Attempts have been made to take this clonal structure into account in association analyses [16], [17], but strong linkage disequilibrium will always restrict the resolution of the approach. To overcome this, studies will require either populations with elevated recombination, a large diverse sample, or both, to make the statistical analysis robust.

The confounding effect from clonal population structure may be less problematic in highly recombinogenic bacteria. Homologous recombination brings genetic admixture into bacterial populations in a manner akin to sexual reproduction in humans, although it does not occur every generation, and only affects a small part of the genome in each occurrence. In *S. pneumoniae*, homologous recombination involves, on average, 2.3 kb of chromosomal DNA [18], about twice the size of an average pneumococcal gene, suggesting that large numbers of recombinational events must accumulate in order to break up linkage blocks smaller than this size. However, the recombination signals left after the action of natural selection are not uniformly distributed across the genome but are concentrated at particular loci, which are commonly known as recombination hotspots. These hotspots are coincident with genes involving the bacterial response to selection pressure, which includes host immune responses and antibiotic utilization, particularly beta-lactams [19], [20]. We hypothesized that the frequency of recombination at these sites would therefore be sufficient to allow the identification of causal SNPs associated with resistance to beta-lactams, given a large enough sample size. Continuing reduction in sequencing costs has allowed the scale of whole-genome bacterial population studies to increase [20], [21], and this should provide more robust statistical power for association analyses. The availability of multiple large bacterial population studies allows a replication of such association studies, providing stronger evidence for common causal SNPs as well as the potential to identify rarer causal SNPs, some of which might only be detected in unique population settings.

Here we conducted an association study using the pneumococcal populations from carriage cohorts in Maela refugee camp, Thailand [20], and Massachusetts, USA [21], two recent species-wide pneumococcal studies from which large numbers of whole genome sequences and phenotypes for beta-lactam susceptibility are available. Given the high recombination frequency in *S. pneumoniae* generally, the observed recombination hotspots covering antibiotic resistance genes, and the relatively large sample sizes of both studies, we hoped to overcome the challenges in performing bacterial association studies discussed above. Therefore this study aimed to more precisely identify the sets of variants associated with resistance, where they were located in the genome, and how they were distributed across the population.

## Results

### Identification of loci associated with beta-lactam non-susceptibility

**Figure 1:**
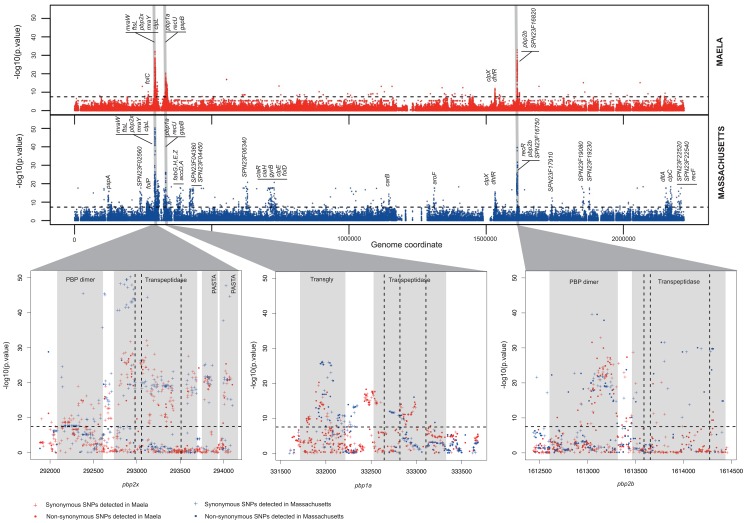
Summary of the genome-wide-association study conducted in two separate datasets. Manhattan plots summarize the association of whole-genome SNP variants with beta-lactam susceptibility in the Maela and Massachusetts data as well as particular gene regions which show strong associations. **Top panel** represents the statistical significance of association (y-axis) for each variant arranged in order on the genome (x-axis) in the Maela (red) and Massachusetts (blue) data. Horizontal dotted lines in both top and bottom panels indicate a significance cut-off after Bonferroni correction of p = 0.01. Genes with significant associations are annotated on top. Genes coding for penicillin binding proteins: *pbp2x*, *pbp1a*, and *pbp2b*, whose roles in beta-lactam resistance are well characterized, are highlighted in grey. **Bottom panel** expands the view of penicillin binding protein genes where most of the significant associations are detected: from left to right: *pbp2x*, *pbp1a*, and *pbp2b*. Protein domains identified within these genes are shaded in pale grey and labelled. The vertical dotted lines represent the active sites of the transpeptidase domain. Plus signs denote synonymous SNPs and dots denote non-synonymous SNPs.

We conducted an association study on whole genome SNPs and insertions or deletions (indels) to identify variants associated with beta-lactam non-susceptibility. Two rounds of analyses were performed separately using 3,085 genomes from pneumococcal strains collected from a carriage cohort in Maela [20], and 616 genomes from a carriage cohort from Massachusetts [21]. Based on the Clinical and Laboratory Standard Institute guidelines (CLSI, 2008), strains with penicillin minimum inhibitory concentration (MIC) ≤0.06 µg/ml were classified as susceptible; applying these cutoffs to our data gave 1,729 case (non-susceptible) and 1,951 control (susceptible) samples for our study (with 21 unknown). The Maela and Massachusetts populations comprise strains from multi-lineage backgrounds. Therefore, taking the population stratification into account is essential to separate the clonal population signals from true phenotypic associations. The population substructures utilized were those defined previously [20], [21], which in both cases were determined using a Bayesian clustering approach (see Methods). Based on this clustering information, the Cochran-Mantel-Haenszel (CMH) association statistic was employed to test for associations between beta-lactam non-susceptibility and specific variants, conditional on the population cluster. For each population, we screened for common alterations with minor allele frequency >0.01 and reported sites with a p value<0.01, incorporating a Bonferroni adjustment for multiple comparisons (**Figure 1** **, Tables S1–S3**).

**Figure 2:**
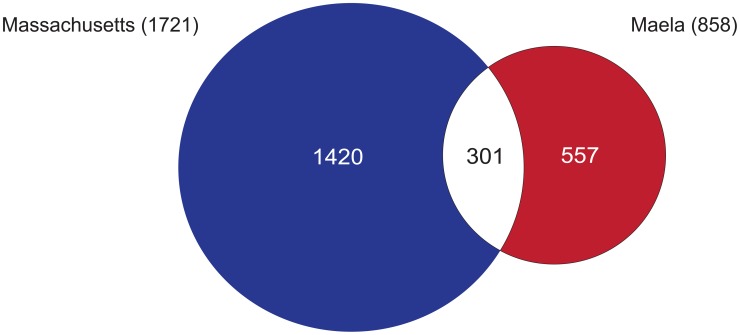
Summary of single nucleotide polymorphisms (SNPs) associated with beta-lactam non-susceptibility. A Venn diagram summarises the number of SNPs reaching significance in each of the Maela and Massachusetts datasets, and those that are co-detected in both.

We found 858 and 1,721 SNPs associated with beta-lactam non-susceptibility in the Maela and Massachusetts populations respectively (**Figure 2** **, Tables S2–S3**). Among these, 301 SNPs were found to be associated with non-susceptibility in both populations. Considering that the two settings have different population structures that have evolved independently, these co-detected SNPs represent a set of candidates in which we can have more confidence. Rather counter-intuitively, more candidate SNPs were identified in the smaller dataset from Massachusetts than in the larger dataset from Maela. There are several potential explanations for this observation; one being that it is due to different linkage structure within the two datasets. Not all of the candidate SNPs will necessarily play a causative role; some may be tightly linked to causative SNPs, with insufficient recombination in the dataset to separate them (here called “hitchhiking” SNPs), and hence form part of the same haplotypes. To test this, we estimated the size of the haplotype blocks that harbor candidate SNPs in both the Maela and Massachusetts populations, using the criteria described in Gabriel *et al.* [22], [23] (see Methods). Haplotype block sizes detected in the Maela data are significantly smaller than the Massachusetts data (Mann Whitney test p value 6.53×10^−9^, **Figure S1**), indicating that many of the candidate SNPs detected in the Massachusetts data are potential hitchhikers, thereby generating some false positive results. The second potential explanation is due to the population stratification defined previously [20], [21]. As the clustering analyses were performed separately on each dataset, it is possible that the clustering information from the two datasets are not equivalent in their stringency, leading to a more strict control over population stratification in one population than the other. Nevertheless, 51 candidate loci, comprising a total of 301 discrete and linked SNPs, were co-detected in both the Maela and Massachusetts data; using these should provide a high-stringency data set that overcomes these population-specific effects. The co-detected loci include three intergenic SNPs, and 298 SNPs detected in coding sequences. The latter can be divided into 71 non-synonymous and 227 synonymous SNPs. Of these 51 loci, nine were single SNPs, and 42 were in linkage blocks of between two and 19 SNPs, of which 12 contained only a single non-synonymous SNP. Based on assembled sequences, we also investigated whether or not indels found in associated genes could contribute to the resistant phenotype. None of the identified indels showed significant association with resistance, after Bonferroni correction, at a p-value of <0.01.

To estimate how much of the phenotypic resistance in our samples could be explained by the identified SNPs, we performed cross-prediction tests using only the SNPs co-detected in both the Maela and Massachusetts association studies, tested back against each population separately. We found that close to 100% of the resistance in each population could be explained by all of the co-detected SNPs (**Figure S2**). Unlike human polygenic traits where each locus contributes only a small effect on the phenotype, each of these bacterial loci appears to have a much stronger effect, and indeed some have been shown experimentally to change the phenotype with only a single variant [1]. This can be demonstrated using odds ratios, which indicate the size of the effect of each associated SNP. While human GWAS studies report a median odds ratio of 1.33 per SNP [24], [25], our analysis gives a median odds ratio of 11.09 per SNP, indicating a stronger effect size. For both the Maela and Massachusetts populations, the percentage of resistance explained plateaued after the addition of approximately 10 loci in any order. This suggests that, at most, about 10 loci are required to make a susceptible strain non-susceptible and that multiple different combinations can achieve this. However, in each resistant isolate, combinations of more than ten loci are commonly detected, perhaps indicating that not all loci are involved in conferring resistance, but that some may play a compensatory role in reducing the fitness cost of resistance variants. In total, the co-detected variants are present in 100% and 98% of the Maela and Massachusetts resistant strains respectively, highlighting that a large proportion of possible resistance variants are captured in our study.

### Biological relevance of candidate loci

For both population settings, loci found to be associated with beta-lactam non-susceptibility show higher enrichment in genes compared to intergenic regions than would be expected by chance (Fisher's Exact Test p-value<0.0001). Candidate loci are not randomly distributed across the whole genome, but clustered within certain genes (**Figure 1**). Co-detected loci in both datasets are localized in genes participating in the peptidoglycan biosynthesis pathway, including penicillin binding proteins (*pbp2x*, *pbp1a*, *pbp2b*) and two transferases required for cell wall biogenesis (*mraW*, *mraY*), the cell division pathway (*ftsL*, *gpsB*), heat shock protein and chaperones (*clpL*, *clpX*), the recombination pathway (*recU*) and a metabolic gene known to confer resistance to co-trimoxazole (*dhfR*). Some of these sites, particularly in the *pbp* genes, matched those previously reported to play an important role in beta-lactam resistance in the literature (**Table S1**). To our knowledge, out of 71 non-synonymous SNPs reported here, 43 SNPs are novel and potentially contribute to beta-lactam non-susceptibility in addition to those identified in previous studies.

### Candidate loci in genes participating in the peptidoglycan biosynthesis pathway

Since most beta-lactam antibiotics work by inhibiting cell wall biosynthesis, it is not surprising to see significant associations between non-susceptible phenotypes and variants in genes participating in the peptidoglycan biosynthesis pathway, including *pbp2x*, *pbp1a*, *pbp2b*, *mraW* and *mraY*. Many single amino acid alterations in *pbp2x*, *pbp1a* and *pbp2b* have been previously demonstrated experimentally to increase pneumococcal resistance to beta-lactams. Mutations within or close to the active sites of the transpeptidase domain in penicillin binding proteins have been reported to be associated with penicillin resistance [26]–[28]. By interfering with the formation of a covalent complex between the active site serine and antibiotic molecules, these mutations help reduce the binding affinity of beta-lactam rings to the transpeptidase enzyme. This allows the pneumococci to form a functional cell wall, and thereby become non-susceptible. We observed many predicted loci co-localizing with or surrounding the transpeptidase active sites. These are recognized as three conserved amino acid motifs, SXXK, SXN and KT(S)G [1], [29] and are highlighted as vertical dotted lines in the bottom panel of **Figure 1**. Many known structurally characterized alterations in *pbp* genes have been rediscovered in our analysis, providing independent validation of some of our results. In *pbp2x*, we observed an association at T338A, which is located next to the active site 337. The side chain of T338 is required for hydrogen-bonding, and the T338A substitution results in a distortion of the active site [1]. In *pbp1a*, an alteration from TSQF to NTGY at position 574, which has been shown to have a lower acylation efficiency *in vitro* [1], [30], was also observed in this analysis. In addition to candidates known to confer structural changes that lead to resistance, we also observed association with E285Q in *pbp1a* which might contribute to a fitness compensation mechanism caused by resistance in *pbp2b* [31]. Other functional conformational changes, as well as variants that matched previous observations from sequence comparison, are tabulated in **Table S1** [1], [2], [5], [6], [30]–[38]. Moreover, we observed substitutions outside *pbp* genes that could potentially affect antibiotic susceptibility, or represent compensatory mutations that interact epistatically with changes associated with resistance. These include the genes *mraY* and *mraW*, which encode transferases. Both function upstream of the *pbp* genes in the peptidoglycan biosynthesis pathway.

### Candidate loci in genes outside the peptidoglycan biosynthesis pathway

The genome-wide screen provided us with an opportunity to identify associations outside the *pbp* genes and the peptidoglycan biosynthesis pathway, which are the direct target of beta-lactams. In both the Maela and Massachusetts datasets, nine independent loci comprising 31 SNPs were detected outside of these pathways. These include amino acid alterations in a major heatshock protein, *clpL*. Mutants lacking *clpL* have been previously reported to be more susceptible to penicillin [1]. The effect was attributed to the ability of *clpL* to interact with and stabilize the Pbp2x protein. In the Massachusetts data alone, we observed associations between resistance and *ciaH*, a histidine kinase sensor, and *ciaR*, its response regulator, consistent with previous studies reporting a large increase in resistance due to *ciaH* mutations. The mutations in the *ciaH* kinase sensor resulted in hyperactivation of the *ciaR* regulator, which in turn leads to increased beta-lactam resistance [1], [39]. Association signals from *ftsL* and *gpsB* genes were detected in both datasets. These two genes function in cell division and are essential for complete cell wall formation. Depletion of GpsB leads to cell deformation with a similar phenotype to that observed when the Pbp2x protein is inhibited by methicillin [40]. These identified candidates potentially interact with *pbp* genes, either directly or indirectly through regulation or participation in cell wall formation; however, experimental characterization will be required to explore the mechanisms of how these alterations influence beta-lactam susceptibility.

### Candidate loci in genes conferring resistance to other antibiotics

Interestingly, strong discrete associations were also found in genes where specific variants are known to confer resistance to co-trimoxazole, an antibiotic targeting the bacterial DNA synthesis pathway [41]. We detected associations in *dhfR* (encoding dihydrofolate reductase) and *folP* (dihydropteroate synthase), which are required for folate synthesis and are essential for nucleotide biosynthesis. Given that beta-lactam and trimethoprim resistance arise from different mechanisms and that the *dhfR* and *folP* loci are not genetically linked to any other detected loci, it is curious as to why we observed these signals. A possible explanation could be the contemporaneous use of both beta-lactam and trimethoprim antibiotics in the host populations studied, which would drive co-selection for resistance to the two unrelated classes of antibiotics. In both the Maela and Massachusetts datasets, strains that are phenotypically resistant to beta-lactams are more likely to be phenotypically resistant to co-trimoxazole, suggesting that the two phenotypes did not occur independently (Fisher's exact test p-value<2.2×10^−16^, **Table 1**). Clinical records from Thailand listed beta-lactams and co-trimoxazole as the first and second most frequently prescribed antibiotics for upper respiratory infection treatments [42], indicating that co-selection pressures may have been possible if the two antibiotics were frequently used together.

**Table 1:** Co-occurrence of co-trimoxazole and beta-lactam resistance phenotypes.

			Beta-lactam		Fisher's exact test p-value and (odds ratio)
			resistant	sensitive	
**Maela**	**Co-trimoxazole**	resistant	1,356	771	<2.2×10^−16^ (10.36)
		intermediate	77	280	
		sensitive	68	517	
**Massachusetts**		resistant	102	38	<2.2×10^−16^ (7.29)
		sensitive	125	341	

Note that the association is significant for the Maela data regardless of how co-trimoxazole resistant and intermediate strains were grouped. Grouping co-trimoxazole intermediate and sensitive strains together still gives a significant association with p-value <2.2×10^−16^ with odds ratio 9.66. Isolates with missing phenotypes were removed from this analysis.

### Beta-lactam specificity of resistance mutations

**Figure 3:**
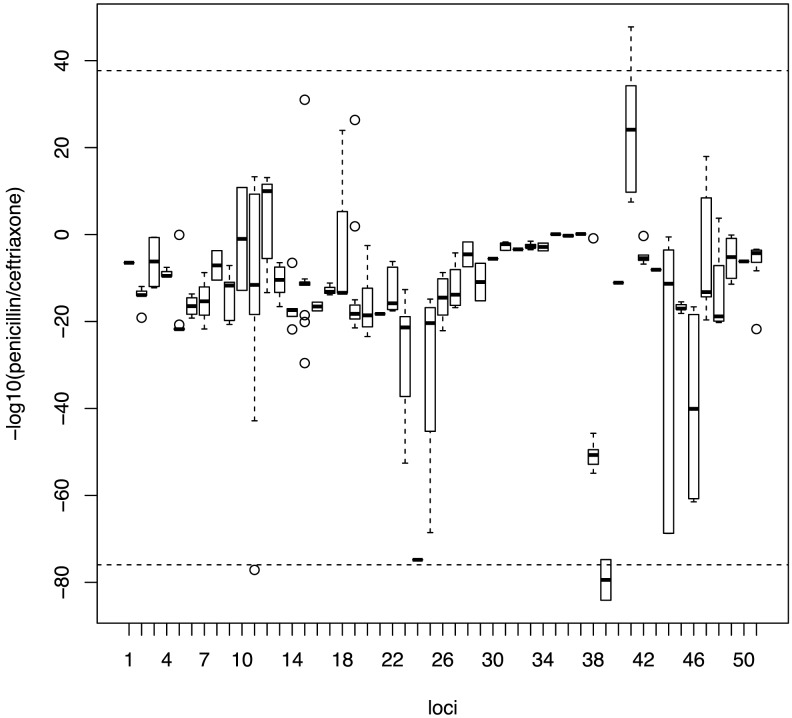
Specificity of association signals for co-detected candidate loci with different classes of beta-lactam antibiotics. Bonferroni-adjusted p-values from associations with continuous phenotypes with each co-detected SNP were grouped into their linkage loci. Positive values on the y-axis show stronger association with penicillin resistance while negative values show stronger association with cephalosporin resistance. Horizontal dotted lines represent the 99^th^ percentile.

As some of the variants detected by our study are known to affect the binding affinity for beta-lactams, we looked to see whether the effect would be equivalent across all classes of beta-lactam antibiotics, or if resistance due to specific variants would be greater for certain classes of antibiotic. To test this, we replicated the analysis on the candidate loci using the continuous phenotype of the minimum inhibitory concentration (MIC) value for two classes of beta-lactam antibiotics; penicillins and cephalosporins (here represented by ceftriaxone). Penicillins and cephalosporins both possess a characteristic beta-lactam ring, but while the beta-lactam ring is fused to a 5-membered thiazolidine ring in penicillins, it is fused to a 6-membered dihydrothiazine ring in cephalosporins. The drugs also differ in side chains that differentiate their kinetic properties [43]. **Figure 3** plots the differential association of each locus to the two beta-lactam antibiotics. Loci with stronger association towards penicillin are distributed along the positive y-axis, while those showing a stronger preference towards cephalosporins are distributed along negative y-axis. We found that loci do not contribute equally to different classes of beta-lactam antibiotics (Kruskal-Wallis rank sum test, p value<2.2×10^−16^), with a strong association of some loci towards resistance to either penicillins or cephalosporins.

### Distribution of candidate alleles in the Maela and Massachusetts populations

Given the known pneumococcal population structure in both the Maela and Massachusetts datasets [20], [21], we sought to explore and compare the prevalence of candidate beta-lactam resistance alleles identified as loci co-detected in the two populations. The two populations are composed of multiple pneumococcal lineages, many of which are present in one population but absent in the other. This difference in population structure has a large influence on the types of resistance alleles detected in each setting. Therefore, an unbiased comparison between the Maela and Massachusetts populations can only be made using the lineages common to both locations. PMEN-14, the globally dispersed multidrug resistant lineage, was detected in both populations (Maela: 2007–2010, Massachusetts 2001–2007), and thus allows a comparative view between the two datasets. PMEN-14 isolates from Maela and Massachusetts have significantly different beta-lactam resistance allelic profiles (Mann-Whitney U test, p value = 4.68×10^−12^).

**Figure 4:**
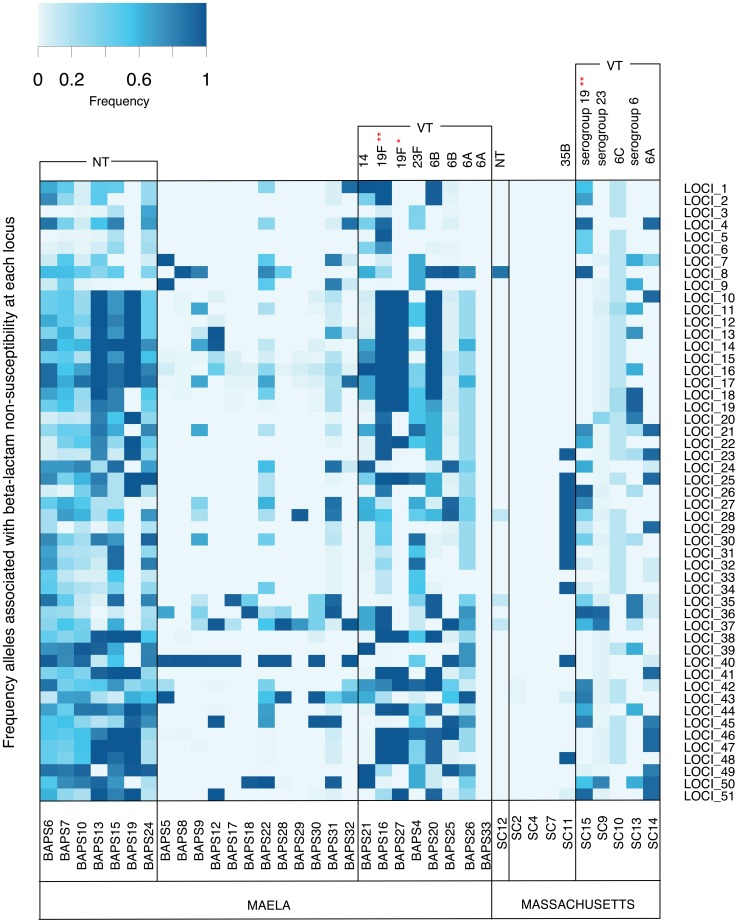
Frequency of putative resistance alleles from candidate loci in the Maela and Massachusetts populations. The resistant allele frequency at each locus (rows) in each population cluster (columns) is represented by different shades on a blue-grey scale. Vaccine and non-vaccine serotype status of each cluster are shown at the top of each column. Asterisks highlight two drug resistant global clones: * represents the PMEN1 lineage; ** represents the PMEN14 lineage. Note that mixed population clusters have been removed from the diagram.

Though the local beta-lactam resistance profiles are different, the pattern of their distribution across the Maela and Massachusetts pneumococci is similar. In both populations, the distribution of resistance alleles is not uniform (**Figure 4**). The multidrug resistant lineages PMEN-14 and PMEN-1, along with other vaccine target lineages appear to carry predicted resistance alleles at a higher frequency. This reflects the vaccine's design to target serotypes associated with antibiotic resistance [21], [44]. However, levels of beta-lactam resistance have generally remained stable post-vaccine introduction [21], [45], [46], which has resulted from the success of resistant non-vaccine lineages with a high frequency of resistance alleles (e.g. 35B in Massachusetts, NT in Maela; **Figure 4**) and serotype switching by resistant-vaccine type lineages such as PMEN-14 [21]. In pneumococcal populations dominated by NT lineages such as in Maela, the higher rate of recombination observed in these lineages [20], and the fact that they are not targeted by current vaccines, may allow them to act as both source and sink for resistance alleles, generating more combinations that are then seeded into the wider population.

## Discussion

The power of phenotype-genotype association studies in bacteria is limited by the clonal population structure and limited recombination within these organisms. One approach to overcoming this is to explicitly account for population structure in the analysis, and this was recently attempted for studies of host-association in *Campylobacter* [16] and *Staphylococcus aureus* [17], but the sensitivity of both was limited by a relatively small sample size. Our analysis used a *S. pneumoniae* data set of much larger size, enhancing statistical power in detecting associated variants. Our approach also took advantage of the higher level of recombination in the genes participating in the peptidoglycan biosynthesis pathway, some of which are known to be recombination hotspots [19], [20] significantly reducing the effect of long haplotype blocks within these important genes. Together, this enabled us to identify specific nucleotide variants underlying beta-lactam resistance in this organism, some of which were previously known, but many of which are novel.

Our analysis allows the refinement of the understanding of resistance beyond “mosaic genes” to identify likely causative variants, and shows that there are multiple loci which may contribute to resistance. We have also been able to show that, while some loci likely contribute universally to all beta-lactam resistance, some can demonstrate a stronger association with resistance to certain classes of antibiotics more than others.

We used this resistance variant dataset to examine the allele distribution within the sampled population. While specific lineages can vary between populations in the resistance loci present, a general finding was that a high frequency of resistance alleles could be found in both vaccine and non-vaccine lineages, a potential explanation for why vaccination has not reduced beta-lactam resistance within the population. Some non-vaccine target lineages with a high frequency of resistant alleles can act as both a source and a sink of resistance alleles within the population.

A limitation of our approach is that it is sensitive to recombination frequency and requires a non-clonal population and a large sample size. Although the recombination frequency of different bacteria is relatively fixed, current sequencing technologies do now allow very high sample sizes within bacterial populations, and this may increase the applicability of this approach in the future. The sensitivity of detection of association will also be enhanced by the occurrence of *de-novo* mutations conferring resistance (homoplasy; [19]), representing convergent evolution. However, this will only be possible using whole-genome sequencing, and these could not be detected by eukaryotic-style marker-SNP-based association studies.

Future use of whole-genome sequencing for antibiotic resistance/sensitivity prediction in clinical practice will rely on the ability to assign function to specific variants, rather than mosaic blocks, and this kind of study will be essential to enable these future applications. Nevertheless, results reported from this genome-wide association study are hypothesis-generating and will require further functional validation.

## Materials and Methods

### Phenotype and genotype data

The test populations represent the largest datasets for which whole genome sequences and antibiotic-resistance phenotypes are available - Maela [20] and Massachusetts [21]. Beta-lactam susceptibilities were determined in both datasets by disk diffusion following the CLSI 2008 guidelines [47]. Our analyses contained 1,501 non-susceptible, 1,568 susceptible and 16 unknown phenotypes in the Maela data; 228 non-susceptible, 383 susceptible and 5 unknown phenotypes from the Massachusetts data. The minimum inhibitory concentrations (MIC) of non-susceptible isolates were confirmed by the E-test method (bioMerieux, Marcy L'Etoile, France). Strain names and a full list of MICs from the Maela dataset [20] are given in **Table S4**. Strains and metadata for the Massachusetts dataset were given as supplementary data in ref [21].

Samples were previously sequenced as multiplexed libraries on Illumina Hiseq 2000 machines using 75-nt or 100 nt paired-end runs as described in [20], [21]. Short reads from both studies have previously been deposited in the European Nucleotide Archive under study number: Maela data - ERP000435, ERP000483, ERP000485, ERP000487, ERP000598 and ERP000599; and Massachusetts data as listed in Table S1 of [21]. Reads from both datasets were mapped onto a single reference genome, *S. pneumoniae* ATCC 700669 (European Molecular Laboratory (EMBL) accession FM211187) [48] using SMALT 0.5.7. (http://www.sanger.ac.uk/resources/software/smalt/). Bases were called from mapped sequences using the methods described in [49], resulting in 392,524 and 198,248 SNP calls from the Maela and Massachusetts data respectively. Genomes from Maela were *de novo* assembled using Velvet [50] with combinations of SSPACE [51], GapFiller [52], BWA [53] and Bowtie [54] as in [20] and genomes from Massachusetts were assembled with Velvet exclusively as described in [21]. Assembled sequences allowed variations from insertions and deletions (indels) to be incorporated for a deeper analysis at each locus.

### Defining population structure

The Maela and Massachusetts populations represent species-wide data sets; they respectively consist of 65 and 46 different capsule types, and at least 277 and 154 known multilocus sequence types. The population substructures as determined in [20] [21] were used in this analysis. Briefly, whole genome-mapped sequences and concatenated core genome sequences were used in the Maela [20] and Massachusetts data [21], respectively, as input to the BAPS software [55]–[57]. BAPS was used to define the clonal population structure by estimating the structure based on non-reversible stochastic optimization. The method has successfully been applied to bacterial populations of several different species [58], [59]. Individual strains in Maela and Massachusetts data were first partitioned into clusters based on multiple runs of the estimation algorithm (Methods in [20] [21]). This resulted in 33 and 16 initial clusters for the Maela and Massachusetts data, respectively. Due to the large sample size of the Maela dataset, BAPS was additionally run in a hierarchical manner. As described in [20], data from each of the primary clusters identified in the Maela data were re-analyzed to obtain secondary clusters within each primary cluster, and these were used to represent the population structure of Maela pneumococci.

### Quality control

The haploid bacterial SNP information was treated as human mitochondrial sequence in PLINK v. 1.07 [60] and controlled for missing rate and allele frequency. We excluded variants with minor allele frequency <0.01, missingness by strain >0.1 and missingness by variants >0.1. For each site, the top two most common variants were parsed to the next analysis to reduce complexity in the test statistic.

### Simulation on case-control association analysis

Intrinsic noise from genetic variation alone can lead to false positive signals. To estimate basal false positive rates and decide a suitable cut-off for each dataset, we separately ran 100 GWAS permutations with true genotypes but randomized binary phenotypes (**Figure S3**). None of the permutations of either the Maela or Massachusetts datasets achieved any significant association at p-value 0.01 with a Bonferroni correction for multiple testing, therefore validating a Bonferroni-adjusted cut-off at p-value 0.01 as our conservative threshold.

### Case-control association analysis with real data

We first determined SNPs associated with beta-lactam resistance with binary phenotypes: susceptible or non-susceptible. However, the intrinsic clonal population structure of bacteria can result in high false positive rate in GWAS. The tests were thus performed conditioned on the population structure generated by BAPS in previous publications [20], [21] and controlled for genomic inflation factor. Based on known cluster information, the Cochran-Mantel-Haenszel (CMH) test for 2×2×K binary phenotype x variants | population cluster was employed with sites corrected for multiple testing using the Bonferroni correction at a p-value of 0.01. The application of the CMH test reduced the genomic inflation factor from 80.16 (mean chi-squared statistic = 68.99) to 2.56 (mean chi-squared statistic = 3.05) in the Maela data, and 13.18 (mean chi-squared statistic = 14.17) to 3.76 (mean chi-squared statistic = 4.73) in the Massachusetts data. The reductions in genomic inflation factor seen in both datasets suggest a decrease in false positive rates due to underlying population structure. However, the genomic inflation factors observed here are relatively high compared to those observed in human nuclear chromosome GWAS, suggesting that intrinsic clonal population structure is still an issue for bacterial association studies.

### Testing the effect of cluster size of the population stratification

Genome wide association studies are sensitive to population stratification. While a stringent stratification helps reduce false positives, it potentially increases false negatives. Due to the size of the Maela dataset, we had available the (more relaxed) primary BAPS clusters, and the (more stringent) secondary BAPS clusters, and we therefore used these to investigate the effect of clustering size with respect to the number of discovered variants and false positive rate in our data. We separately repeated the Cochran-Mantel-Haenszel (CMH) test as described above using information on primary and secondary BAPS clusters as previously defined [20]. We detected greater numbers of variants with significant associations when stratified by primary clusters compared to secondary clusters (10,451 SNPs compared to 858 SNPs). Also, a higher false positive rate was observed in the analyses using the primary clusters than the secondary clusters (genomic inflation factor of 6.58 compared to 2.56). This result is consistent with what is expected, reflecting a trade-off between false positives and false negatives, and will be dependent on the sample size and underlying population structure.

### Linkage analysis

A high genomic inflation factor indicated that some of the candidate alleles were influenced by population structure and were likely to be hitchhikers. We explicitly tested for linkage disequilibrium between candidate SNPs using Haploview version 4.2 [61]. The information was treated as male human X-chromosome to retain its haploidy. Haploview was devised for human genetics where linkages between distant sites are disrupted by crossing-over. Unlike human, bacterial recombination does not necessarily break long distance linkage. We therefore set Haploview to consider all pairwise comparisons under 2,200 kb, which is the size of the whole *S. pneumoniae* genome, thus incorporating all possible linkage predictions into our analysis. Using 95% confidence bounds as described in [22], a haplotype block was identified as a region with a low recombination rate (**Figure S4)**. These linkage blocks were used to show the context of the predicted alleles and thus potential limitations of our study. We also compared physical linkage size (**Figure S1**) detected in the Maela and Massachusetts data. The smaller linkage blocks found in the Maela data suggest a higher likelihood of capturing recombination in the larger dataset and thus separating causative SNPs from hitchhiking SNPs.

### Estimation of percent resistance in the population that can be explained by the candidate loci

We plotted the proportion of resistance in the population that could be explained by the co-detected loci in each of the test populations, using only combinations of variants observed in both the Maela and Massachusetts datasets (**Figure S2**). The order of loci added was permutated to accommodate all possible combinations.

### Specificity to different classes of beta-lactams

To test whether or not there were variants conferring more specific resistance to certain classes of beta-lactam antibiotics, we repeated the analysis on co-detected candidate SNPs in both datasets and replaced the binary phenotypes with continuous phenotypes: penicillin MIC values and ceftriaxone MIC values. P-values calculated from penicillin MIC and ceftriaxone MIC for each SNP were grouped by the linkage structure.

### Prevalence of candidate loci in the population

For each BAPS cluster in both the Maela and Massachusetts data, we calculated the mean prevalence of candidate loci by averaging the frequency of linked SNPs detected in each locus per cluster size.

All statistical analysis were performed in PLINK version 1.07 and R version 2.11.1. Graphical representations were created in R.

## Supporting Information

Figure S1 Summary of physical linkage structure in two separate datasets. Size of linkage loci detected in the Maela (red) and Massachusetts (blue) association studies were plotted as histograms on log10 scale. Vertical dotted lines mark the median size of haplotype blocks that harbor candidate SNPs (37.5 bp in the Maela data and 165 bp in the Massachusetts data).
(EPS)

Figure S2 Percentage of the non-susceptible phenotype explained by co-detected loci in the Maela and Massachusetts populations. The plots represent proportions of resistance in the population (y-axis) explained by all combinations of increasing numbers of co-detected loci (x-axis), based on combinations of loci observed from both the Maela and Massachusetts data.
(EPS)

Figure S3 Randomised control for intrinsic noise based on genetic variation alone. Manhattan plots (see legend to Fig. 1) demonstrate no significant associations in either Maela or Massachusetts data using real genotype data and randomized resistance phenotype assignments. Horizontal dotted lines mark the cutoff with Bonferroni correction at p = 0.01.
(EPS)

Figure S4 Linkage analysis for variants co-detected in two separate datasets. The Haploview plot illustrates linkage disequilibrium (r^2^) between co-detected SNP candidates from the Maela and Massachusetts datasets. The bar on the left represents the genome position of the SNPs, connected by lines to the diamond plot, right. A complete black diamond represents complete linkage disequilibrium between candidate SNPs (r^2^ = 1), while a white diamond represents a perfect equilibrium (no linkage) (r^2^ = 0).
(EPS)

Table S1 Associations co-detected in two separate datasets. The table summarizes all association statistics, linkage disequilibrium (LD) analysis, biological relevance and literature references for co-detected SNPs and associated loci in the Maela and Massachusetts data. From left to right, the columns represent coordinates from the reference genome (*S. pneumoniae* ATCC 700669), coding regions in which associations were detected, putative resistance nucleotide alleles, putative sensitivity nucleotide alleles, minor allele frequency (MAF), odds ratios (OR), synonymous and non-synonymous changes, positions on protein sequences with observed amino acid alterations, amino acid residues, alternative amino acid residues, literature reports, PubMed identifier (PMID), and linkage information.
(XLS)

Table S2 Associations detected in the Maela data. The table summarizes all association statistics and linkage disequilibrium (LD) analyses for variations that show significant associations in the Maela data. From left to right, the columns represent coordinate from the reference genome (*S. pneumoniae* ATCC 700669), majority detected SNPs, minor allele frequency, minor detected SNPs, odd ratios, linkage information and gene information.
(XLS)

Table S3 Associations detected in the Massachusetts data. Summary of all association statistics and LD analysis for variations that show significant association in Massachusetts data. Columns were summarized using the same scheme described in Table S2.
(XLS)

Table S4 Additional strain information for beta-lactam susceptible and non-susceptible phenotypes for the Maela dataset. Susceptibilities were determined by disk diffusion with oxacillin (1 µg oxacillin disk zone diameter of <20 mm). Minimum inhibitory concentration (MIC) values were determined by E-test if disk diffusion test indicated potential penicillin non-susceptibility [20].
(XLS)
